# HIV drug resistance patterns in pregnant women using next generation sequence in Mozambique

**DOI:** 10.1371/journal.pone.0196451

**Published:** 2018-05-09

**Authors:** María Rupérez, Marc Noguera-Julian, Raquel González, Sonia Maculuve, Rocío Bellido, Anifa Vala, Cristina Rodríguez, Esperança Sevene, Roger Paredes, Clara Menéndez

**Affiliations:** 1 Manhiça Health Research Center (CISM), Manhiça, Maputo, Mozambique; 2 ISGlobal, Barcelona Ctr. Int. Health Res. (CRESIB), Hospital Clínic—Universitat de Barcelona, Barcelona, Spain; 3 Consorcio de Investigación Biomédica en Red de Epidemiología y Salud Pública (CIBERESP), Madrid, Spain; 4 IrsiCaixa AIDS Research Institute, Badalona, Catalonia, Spain; 5 Universitat de Vic-Universitat Central de Catalunya, Vic, Catalonia, Spain; 6 Faculdade de Medicina, Universidade Eduardo Mondlane (UEM), Maputo, Mozambique; 7 Lluita Contra la Sida Foundation, HIV Unit, Hosp Univ Germans Trias i Pujol, Badalona, Catalonia, Spain; Universita degli Studi di Roma Tor Vergata, ITALY

## Abstract

**Background:**

Few data on HIV resistance in pregnancy are available from Mozambique, one of the countries with the highest HIV toll worldwide. Understanding the patterns of HIV drug resistance in pregnant women might help in tailoring optimal regimens for prevention of mother to child transmission of HIV (pMTCT) and antenatal care.

**Objectives:**

To describe the frequency and characteristics of HIV drug resistance mutations (HIVDRM) in pregnant women with virological failure at delivery, despite pMTCT or antiretroviral therapy (ART).

**Methods:**

Samples from HIV-infected pregnant women from a rural area in southern Mozambique were analysed. Only women with HIV-1 RNA >400c/mL at delivery were included in the analysis. HIVDRM were determined using MiSeq® (detection threshold 1%) at the first antenatal care (ANC) visit and at the time of delivery.

**Results:**

Ninety and 60 samples were available at the first ANC visit and delivery, respectively. At first ANC, 97% of the women had HIV-1 RNA>400c/mL, 39% had CD4+ counts <350 c/mm^3^ and 30% were previously not on ART. Thirteen women (14%) had at least one HIVDRM of whom 70% were not on previous ART. Eight women (13%) had at least one HIVDRM at delivery. Out of 37 women with data available from the two time points, 8 (21%) developed at least one new HIVDRM during pMTCT or ART. Twenty seven per cent (53/191), 32% (44/138) and 100% (5/5) of the mutations that were present at enrolment, delivery and that emerged during pregnancy, respectively, were minority mutations (frequency <20%).

**Conclusions:**

Even with ultrasensitive HIV-1 genotyping, less than 20% of women with detectable viremia at delivery had HIVDRM before initiating pMTCT or ART. This suggests that factors other than pre-existing resistance, such as lack of adherence or interruptions of the ANC chain, are also relevant to explain lack of virological suppression at the time of delivery in women receiving antiretrovirals drugs during pregnancy.

## Introduction

Women are disproportionately vulnerable to HIV infection, particularly in sub-Saharan Africa (SSA), where approximately three-quarters of new infections are in women of reproductive age [1]. HIV infection in pregnancy has important public health consequences; it is responsible for the majority of new HIV pediatric infections and a significant cause of maternal mortality [2, 3]. Mother to child transmission of HIV (MTCT) can occur during pregnancy, labor and through breastfeeding [4, 5]. Among the many factors that have been suggested to increase the risk of MTCT, maternal HIV viral load at delivery has been shown to be the strongest predictor [6, 7]. Provision of antiretroviral (ARV) drugs in pregnancy is the most effective intervention for blocking viral replication and reducing MTCT and HIV/AIDS maternal related deaths [1, 8]. However, in low-income countries, increasing occurrence of HIV drug resistance mutations (HIVDRM) to first line recommended ARV drugs either for prevention of MTCT (pMTCT) or antiretroviral therapy (ART) and cross resistances to second line ARV drugs have been found [9]. Pre-existence of transmitted drug resistant viruses or selection of ARV drug resistance during pregnancy, may hinder the long-term efficacy of MTCT preventive programs, as well as severely constrain future treatment options for both mother and child.

Mozambique is one of the 10 countries with the highest HIV prevalence in the world [10]. A study showed that 90% of the subjects on ART with virological failure had developed resistance to first-line treatment options [11]. In addition, a prevalence of 12% of acquired HIVDRM was reported in HIV infected Mozambican children [12]. However, little information on HIV resistance in pregnancy has been reported in the country. As ART coverage continues to grow, better knowledge of the effectiveness of first line ARV drugs in HIV-infected pregnant women in Mozambique is key for antenatal care (ANC) and pMTCT programme optimization and planning. We describe here the frequency and characteristics of HIVDRM, at first ANC visit and at the time of delivery, in pregnant women participating in a clinical trial on malaria prevention in Southern Mozambique.

## Materials and methods

### Study design

This is an observational study to describe the prevalence of HIVDRM at first ANC visit and at delivery in HIV-infected pregnant women, who were enrolled, from March 2010 to April 2012, in a randomized controlled clinical trial to evaluate the safety and efficacy of MQ compared to placebo for malaria prevention (ClinicalTrials.gov, NCT0081121, and Pan African Clinical Trials, PACTR2010020001429343) [13].

### Study area and population

The study was carried out in Manhiça, a semi-rural area in southern Mozambique. Since 1996, the Centro de Investigaçao em Saúde de Manhiça (CISM) is conducting a continuous demographic surveillance in the Manhiça District that covers a population of approximately 95,000 inhabitants in an area of 500km.^2^ [14]. Although the countrywide HIV average prevalence figure is 11% a community based study in adults undertaken in the Manhiça area in 2012 reported nearly 40% of HIV prevalence, while 30% of pregnant women attending the ANC were HIV seropositive [15, 16]. The predominantly subtype in the area is subtype C [17, 18]. At the time of this study, pMTCT relied on ARV prophylaxis, which consisted in antepartum monotherapy with zidovudine (AZT), a single dose nevirapine (sd NVP) at the onset of labor, and AZT + lamivudine (3TC) at delivery and for 7 days postpartum. National guidelines recommended ART to HIV-infected pregnant women for their own health if CD4+T cell count were <350 and/or 3–4 HIV/AIDS WHO clinical stage. The recommended first-line regimen consisted in the dual NRTI backbone (3TC and either AZT or stavudine [d4T]) plus an NNRTI (either NVP or efavirenz [EFV]). However, in 2013, Mozambique adopted “Option B+”, consisting in the initiation of lifelong ART with tenofovir (TDF) + 3TC + EFV in all HIV-positive pregnant and lactating women regardless of their immune status and clinical stage [19]. Both currently and at the time of the study, to assess intra-utero and perinatal MTCT national guidelines recommend to perform an HIV-DNA PCR test between 4–6 weeks of age in all infants born to HIV-infected mothers, with a confirmatory test on a new sample in those infants who test positive.

### Study procedures

#### Enrolment of pregnant women

Pregnant women attending the ANC clinics for the first time were screened for eligibility to participate in the trial. Inclusion criteria were, permanent residency in the area, a gestational age ≤28 weeks, a positive HIV test, absence of history of allergy to intervention drugs, absence of history of severe renal, hepatic, psychiatric, or neurological disease, and no MQ or halofantrine treatment in the preceding four weeks. Following national guidelines, HIV status was assessed after voluntary HIV counseling. Hemoglobin (Hb) and the syphilis rapid plasma reagin test (RPR) were also assessed at this visit as part of routine ANC. Socio-demographic and clinical characteristics were recorded on standardized questionnaires and five mL of venous blood was collected for CD4+T cell count and HIV viral load determination. Administration of ARV drugs for pMTCT or for ART was registered in the study concomitant medication forms.

Following physical examination, enrolled women with gestational age ≥13 weeks received the first administration of IPTp (either placebo or MQ) under supervision. The second and third administrations of IPTp-MQ/placebo were given in the next ANC visits at least one month apart. All women also received study CTXp tablets for daily prophylaxis.

#### Follow-up of pregnant women and their children

Women were followed-up through study visits during pregnancy, delivery and up to one month post-partum. At delivery, a sample from the mother’s peripheral blood was collected for Hb, CD4+T cell count, HIV viral load and malaria infection evaluation. Following national guidelines for pMTCT of HIV, a capillary blood sample was collected from the infant at six weeks of age for HIV PCR analysis to assess vertical transmission.

### Laboratory methods

HIV serostatus was assessed using rapid test (Determine, Abbot Laboratories, USA), and positive results confirmed using Unigold rapid test (TM HIV, Trinity Biotech, Ireland), following national guidelines. HIV-1 RNA levels were determined from cryopreserved plasma samples using the COBAS AMPLICOR or AmpliPrep (Roche Diagnostics, Rotkreuz, Switzerland) devices; these assays have a lower detection limit ranging from 50 to 400 copies/mL. CD4+ T cell counts were determined by flow cytometry after staining of whole blood for CD3, CD8, CD4 and CD45 using fluorochrome-labelled antibodies and acquisition using FACSCalibur (BD Biosciences) and Trucount tubes (Becton Dickinson, San Jose, CA, USA). Remaining plasma samples from first ANC and delivery visits were stored at the CISM laboratory at -80 C°. Analysis of genotypic drug resistance of HIV in stored plasma samples was attempted in all subjects who had HIV-1 RNA ≥400 copies/mL at delivery. HIV-1 RNA was extracted from 140 mL of plasma (QIAamp Viral RNA Mini Kit,Qiagen). Pol amplicons (PR/RT) were PCR-generated, fragmented using Nextera-XT and sequenced in an Illumina MiSeq^TM^ [20]. Next-Generation Sequencing (NGS) raw data was analyzed using an in-house pipeline (https://www.paseq.org). Briefly, we filtered low quality and potentially contaminant sequences and aligned them against a user-specified reference (NCBI Accesion: 97ZA012) using BWA. Codon-level variant calling was then performed on the resulting alignments and an amino acid variant table was generated using a 1% user specified detection threshold with a median of 5147 depth of coverage (IQR: 3295–7281). Drug resistance-associated mutations in protease and reverse transcriptase gene regions from all quality-assured sequences were interpreted with the Standford HIV database (HIVdb) program. To predict susceptibility to NRTIs, NNRTIs and PIs a resistance score was calculated as susceptible, intermediate level and high level following the Stanford HIVdb scoring system (http://hivdb.stanford.edu).

### Data management, statistical methods, and definitions

Data were double-entered using the OpenClinica Enterprise^TM^ software for clinical data management (www.openclinica.com).

Literacy was defined as knowing how to read and/or write. Adolescent women were considered those <19 years of age. CD4+T cell count was categorized as < 350 or > = 350 cells/μl. Detectable viral load was defined as HIV-1 RNA levels ≥400copies/mL. HIV drug resistance was considered as the presence of one or more major resistance mutations as defined by the Stanford HIVdb.

Baseline characteristics of study patients were described using standard statistics. Proportions for categorical variables were assessed using the Chi-squared test or Fisher’s exact test where appropriate. Student's t-test or the Mann–Whitney test was used for comparing means and medians of quantitative variables according to variable characteristics. All statistical tests were two-tailed and statistical significance was defined as p<0.05. Data analysis was performed using Stata version 13 (Stata Corp., College Station, TX, US).

### Ethics statement and participants’ safety

This is an exploratory analysis nested in a clinical trial, which protocol and informed consent forms were reviewed and approved by the Ethics Committees from the Hospital Clínic of Barcelona (Spain) and the local regulatory authorities and National Ethics Review Committee from Mozambique (209/CNBS/2014). Study participants signed a written informed consent form prior to enrolment. The study was conducted under the provisions of the Declaration of Helsinki and in accordance with Good Clinical Practices guidelines set up by the WHO and by the International Conference of Harmonization. The protocol for the HIV resistance analysis in samples of women participating in the trial was reviewed and approved by the National Ethics Review Committee from Mozambique.

## Results

### Study profile

**Fig 1:**
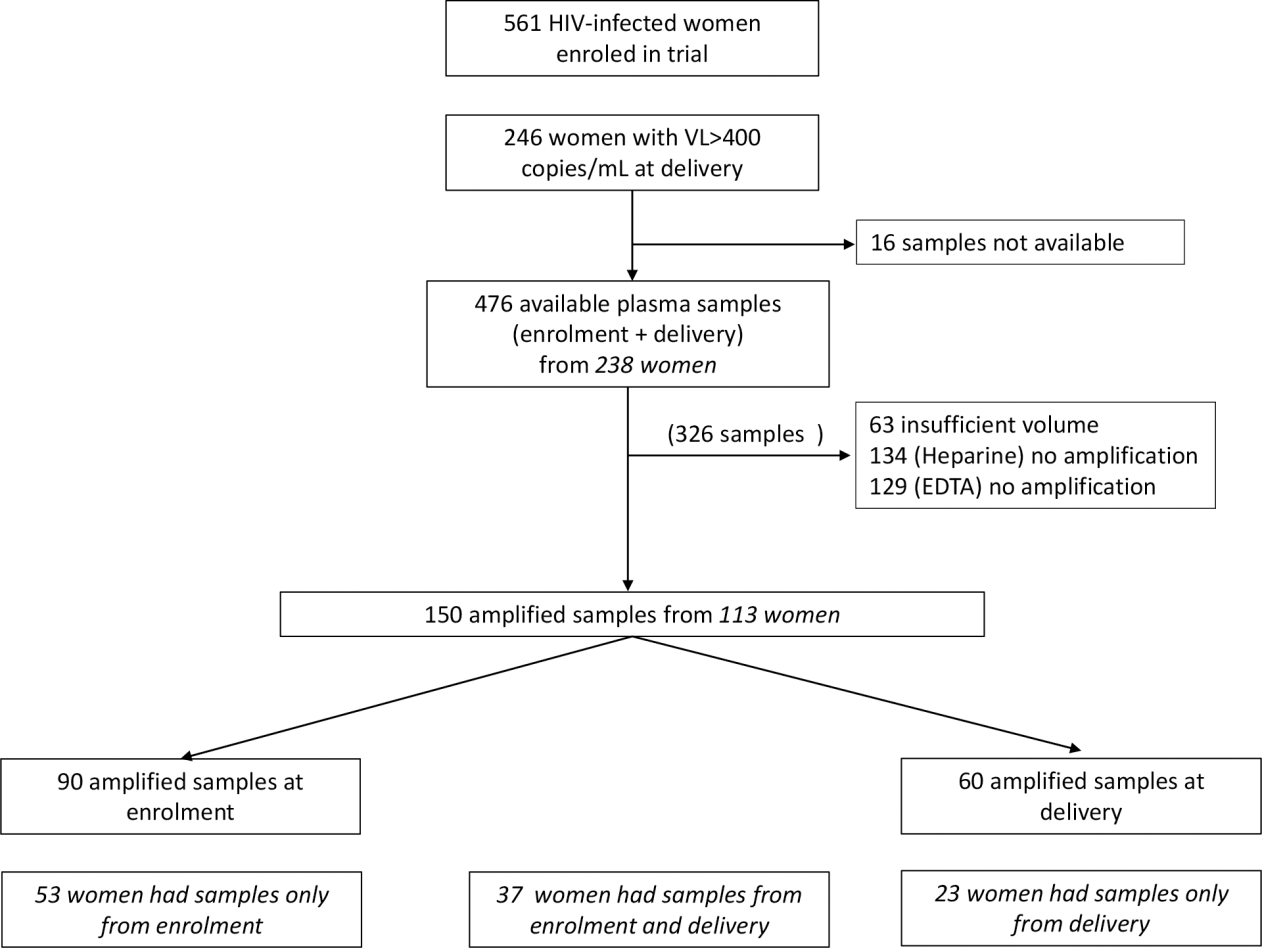
Study profile.

Out of 561 enrolled HIV-infected pregnant women, 246 had detectable viral load (HIV-1 RNA ≥400 copies/ml) at delivery. Out of these 246 women, there were available stored plasma samples from the first ANC and the delivery from 238 women. Out of these 476 plasma samples, 63 had insufficient plasma volume, and in 263 plasma samples RNA could not be amplified. Overall, RNA was successfully amplified in samples from 113 women, and were thus, the study population for this exploratory analysis; 53 women had samples amplified only at enrolment, 23 only at delivery and 37 at both time points (Fig 1).

Out of these 113 women, 18 (16%) were adolescents, 33 (29%) were illiterate and the median gestational age was 22 weeks, Interquartile Range, (IQR) 17–24. Twenty three (20%) of the women were primigravidae, 44 (39%) had CD4+ T-cell counts <350 cells/mm^3^ and almost all [109 (96%)] had detectable viral load at enrolment. Thirty five women (31%) were on previous ART with first line regime at the time of enrolment (Table 1).

**Table 1:** Baseline characteristics at enrolment of study participants (N = 113).

Characteristics	n	%
Age, median (IQR)	25 [21–30]	
Adolescents^1^	18	15.93
Gestational age, median (IQR)^2^	22 [17–24]	
3^rd^ trimester	10	8.85
Primigravidae	23	20.35
Number of previous pregnancies, median (IQR)	2 [1–3]	
Illiteracy^3^	33	29.20
HIV-1 RNA (copies/mL) median (IQR)	35660 [9821–118467]	
Detectable HIV-1 RNA ^4^	109	96.46
CD4+ T-cell counts (cell/mm^3^), median (IQR)	342 [196–559]	
CD4+ T-cell counts <350 cell/mm^3^	44	38.94
Previously on antiretroviral therapy	35	30.97

^1^ Age<19 years

^2^ Gestational age calculated from fundal height

^3^ Not being able to read and/or write

^4^ HIV-1 RNA>400 copies/ml

### HIV drug resistance mutations at enrolment

Ninety pregnant women at enrolment were included in this part of the analysis. The median age was 25 years (IQR 21; 29), and the median gestational age 21 weeks (IQR 16; 23). Twenty one (23%) women were primigravidae, 29 (32%) were illiterate and 15 (17%) were adolescents.Nearly all women [87 (97%)] had detectable viral load with a median viral load of 47,877 copies/ml (IQR 12,807–140,129) In 35 (40%) of them, CD4+ T-cell counts were <350 cells/mm^3^. Only 27 women (30%) were on ART at the time of the first ANC (Table 2). Thirteen (14%) presented at least one HIVDRM, 6 (7%) had resistance to NNRTI, 8 (9%) to NRTI and 2 (2%) to PI. Among the women with at least one HIVDRM, seven (8%) had resistant mutations to at least one drug of the first-line regimen recommended at the time of the study (Table 3). Among the women with HIVDRM to NRTI, 4 (4%) had resistant mutations to ddI, 4 (4%) to TDF and 4 (4%) to 3TC. Of the women who had resistant mutations to NNRTI, 6 (7%) were to EFV, 6 (7%) to NVP and 2 (2%) to RPV (Table 4). The most frequent mutations are shown in Table 5. No significant differences were observed in baseline characteristics between women who presented at least one HIVDRM and those who did not (Table 2).

**Table 2:** Characteristics of women at enrolment and at delivery.

Characteristics	Enrolment							Delivery						
	All (N = 90)		At least one HIVDRM^1^(N = 13)				No HIVDRM^1^(N = 77)			P-value	All (N = 60)		At least one HIVDRM^1^ (N = 8)		No HIVDRM^1^ (N = 52)		P-value
	n	%	n	%	n	%	n	%	n		%	n	%				
**Characteristics at enrolment**														
**Age**^**2**^^,^^**3**^	25 [21–29]		26 [19–29]		25 [21–29]		0.895	26 [22–30]		28 [25–31]		26 [22–30]		0.290
**Adolescents**^**3**^	15	16.67	3	23.08	12	15.58	0.449	9	15.00	0	0.00	9	17.31	0.339
**Gestational age**^**2**^^,^^**4**^	21 [16–23]		20 [12–22]		21 [16–23]		0.468	22 [18–25]		21 [18–25]		22 [19–25]		0.819
**3**^**rd**^ **trimester**	19	21.11	2	15.38	17	22.08	0.728	16	26.67	2	25.00	14	26.92	1.000
**Primigravidae**	21	23.33	3	23.08	18	23.38	1.000	12	20.00	2	25.00	10	19.23	0.655
**Number of previous pregnancies**^**2**^	2 [1–3]		1 [1–3]		2 [1–2]		0.879	2 [1–3]		1 [1–5]		2 [1–3]		0.885
**Illiteracy**^**5**^	29	32.22	5	38.46	24	31.17	0.749	17	28.33	3	37.50	14	26.92	0.676
**HIV-1 RNA (copies/mL)**^**2**^	47877 [12807–140129]		48486 [26200–166409]		47268 [12105–116933]		0.578	32124 [6990–107693]		208266 [16442–425395]		30500 [6990–72500]		0.106
**Detectable HIV-1** ^**6**^	87	96.66	13	100.00	74	96.10	1.000	3	5.00	1	12.50	2	3.85	0.065
**CD4+ T-cell counts (cell/mm**^**3**^**)**^**2**^	402 [255.5–595.5]		432 [194–576]		401 [277–596]		0.666	419 [269–654]		226 [116–285]		440 [295–664]		0.010
**CD4+ T-cell counts <350 (cell/mm**^**3**^**)**	35	38.89	5	38.46	30	38.96	1.000	23	38.33	7	87.50	16	30.77	0.008
**Previous antiretroviral therapy**	27	30.00	4	30.77	23	29.87	1.000	17	28.33	6	75.00	11	21.15	0.009
**Characteristics at delivery**														
**IPTp with MQ in pregnancy** ^**7**^^,^^**8**^	42	46.67	5	38.46	37	48.05	0.564	28	46.67	4	50.00	24	46.15	1.000
**HIV-1 RNA (copies/mL)**^**2**^	35028 [7077–81277]		40053 [26200–60319]		29945 [6358–81443]		0.667	34943 [12891–78116]		155732 [37537–253078]		30500 [6990–72500]		0.024
**CD4+ T-cell counts (cell/mm**^**3**^**)**^**2**^	348 [193–566]		333 [186–559]		349 [193–584]		0.919	379 [250–584]		129 [71–5419]		440 [295–664]		0.071
**CD4+ T-cell counts <350 cell/mm**^**3**^ **(cell/mm**^**3**^**)**	40	44.44	6	46.15	34	44.16	1.000	25	41.67	5	62.50	19	38.46	0.259
**ARV during pregnancy: -PMTCT**^**9**^	73	81.11	8	61.54	65	84.42	0.057	46	81.67	4	50.00	42	84.00	0.018
- **ART**^**10**^	9	10.00	2	15.38	7	9.09		8	11.67	4	50.00	4	8.00	
- **None**	8	8.89	3	23.08	5	6.49		4	6.67	0	0.00	4	8.00	
**MTCT** ^**11**^^,^^**12**^	8	9.20	2	16.67	6	8.00	0.687	4	8.51	0	0.00	4	9.52	1.000

^1^Drug resistance mutation

^2^ Median (IQR)

^3^ Age<19 years

^4^ Gestational age calculated from fundal height

^5^ Not being able to read and/or write

^6^ HIV-1 RNA>400 copies/mL

^7^Intermittent treatment of malaria in pregnancy

^8^Mefloquine

^9^ Prevention of mother to child transmission

^10^Antiretroviral therapy

^11^ Mother to child transmission of HIV

^12^ Data available from 87 women at enrolment and from 47 at delivery

**Table 3:** Frequency of resistance mutations by drug category at enrolment, at delivery and acquired during pregnancy.

Resistance category	Enrolment (N = 90)		Delivery (N = 60)		Acquired during pregnancy (N = 37)	
	n	%	n	%	n	%
At least one DRM^1^	13	14.4	8	13.3	5	13.5
At least one NNRTI DRM^2^	6	6.7	5	8.3	2	5.4
At least one NRTI DRM^3^	8	8.9	6	10.0	2	5.4
At least one PI DRM^4^	2	2.2	0	0.0	1	2.7
At least one drug in first-line regime	7	7.8	6	10.0	1	2.7

^1^ Drug resistance mutation

^2^ Non-nucleoside reverse-transcriptase inhibitors

^3^ Nucleoside reverse-transcriptase inhibitors

^4^ Protease inhibitors

**Table 4:** Predicted drug susceptibility in women at enrolment and at delivery.

Drug type	Enrolment (N = 90)				Delivery (N = 60)		
	Drug	S^1^	I^2^	R^3^	S	I	R
**NRTI**^**4**^	**ABC**	82 (91.1%)	8 (8.9%)	0 (0%)	54 (90.0%)	5 (8.3%)	1 (1.7%)
	**AZT**	87 (96.7%)	3 (3.3%)	0 (0%)	58 (96.7%)	2 (3.3%)	0 (0%)
	**DDI**	86 (95.6%)	0 (0%)	4 (4.4%)	56 (93.3%)	1 (1.7%)	3 (5.0%)
	**D4T**	83 (92.2%)	7 (7.8%)	0 (0%)	56 (93.3%)	3 (5.0%)	1 (1.7%)
	**FTC**	82 (91.1%)	4 (4.4%)	4 (4.4%)	55 (91.7%)	2 (3.3%)	3 (5.0%)
	**TDF**	86 (95.6%)	0 (0%)	4 (4.4%)	58 (96.7%)	0 (0%)	2 (3.3%)
	**3TC**	82 (91.1%)	4 (4.4%)	4 (4.4%)	55 (91.7%)	2 (3.3%)	3 (5.0%)
**NNRTI**^**5**^	**EFV**	84 (93.3%)	0 (0%)	6 (6.7%)	54 (90.0%)	2 (3.3%)	4 (6.7%)
	**ETR**	87 (96.7%)	3 (3.3%)	0 (0%)	57 (95.0%)	1 (1.7%)	2 (3.3%)
	**NVP**	82 (91.1%)	2 (2.2%)	6 (6.7%)	52 (86.7%)	3 (5.0%)	5 (8.3%)
	**RPV**	79 (87.8%)	9 (10.0%)	2 (2.2%)	51 (85.0%)	7 (11.7%)	2 (3.3%)
**PI**^**6**^	**ATV/r**	89 (98.9%)	1 (1.1%)	0 (0%)	60 (100%)	0 (0%)	0 (0%)
	**DRV/r**	89 (98.9%)	1 (1.1%)	0 (0%)	60 (100%)	0 (0%)	0 (0%)
	**FPV/r**	86 (95.6%)	3 (3.3%)	1 (1.1%)	60 (100%)	0 (0%)	0 (0%)
	**IDV/r**	86 (95.6%)	4 (4.4%)	0 (0%)	60 (100%)	0 (0%)	0 (0%)
	**LPV/r**	86 (95.6%)	4 (4.4%)	0 (0%)	60 (100%)	0 (0%)	0 (0%)
	**NFV**	69 (76.7%)	20 (22.2%)	1 (1.1%)	48 (80.0%)	12 (20.0%)	0 (0%)
	**SQV/r**	86 (95.6%)	4 (4.4%)	0 (0%)	60 (100%)	0 (0%)	0 (0%)
	**TPV/r**	88 (97.8%)	2 (2.2%)	0 (0%)	60 (100%)	0 (0%)	0 (0%)

^1^ Intermediate resistance

^2^ Resistant

^3^ Susceptible

^4^ Nucleoside reverse-transcriptase inhibitors

^5^Non-nucleoside reverse-transcriptase inhibitors

^6^ Protease inhibitors

**Table 5:** HIV resistant mutations in HIV-infected pregnant women at enrolment, at delivery and acquired during pregnancy.

Type of mutation	Enrolment (N = 90)		Delivery (N = 60)		Acquired during pregnancy (N = 37)	
	All mutations	*Minority* *Mutations* *(freq <20%)*	All mutations	*Minority* *mutations* *(freq <20%)*	All mutations	*Minority* *mutations* *(freq <20%)*
	N (%)	*n (%)*	N (%)	*n (%)*	N (%)	*n (%)*
**PR mutations**^**1**^						
A71T	7 (7.78)	*3 (42*.*86)*	2 (3.33)	*0 (0)*	0 (0)	*0 (0)*
A71V	3 (3.33)	*2 (66*.*67)*	0 (0)	*0 (0)*	0 (0)	*0 (0)*
D60E	20 (22.22)	*3 (15*.*00)*	12 (20.00)	*1 (8*.*33)*	0 (0)	*0 (0)*
G16E	11 (12.22)	*4 (36*.*36)*	11 (18.33)	*4 (36*.*36)*	0 (0)	*0 (0)*
I62V	12 (13.33)	*2 (16*.*67)*	6 (10.00)	*1 (16*.*67)*	0 (0)	*0 (0)*
I64L	4 (4.44)	*0 (0)*	2 (3.33)	*0 (0)*	0 (0)	*0 (0)*
I64V	2 (2.22)	*2 (100*.*00)*	0 (0)	*0 (0)*	0 (0)	*0 (0)*
K20M	3 (3.33)	*2 (66*.*67)*	0 (0)	*0 (0)*	0 (0)	*0 (0)*
K20R	32 (35.56)	*3 (9*.*38)*	24 (40.00)	*1 (4*.*17)*	1 (2.70)	*0 (0)*
L10I	5 (5.56)	*2 (40*.*00)*	3 (5.00)	*1 (33*.*33)*	0 (0)	*0 (0)*
L10V	2 (2.22)	*0 (0)*	3 (5.00)	*1 (33*.*33)*	0 (0)	*0 (0)*
L33V	1 (1.11)	*0 (0)*	1 (1.67)	*1 (100*.*00)*	0 (0)	*0 (0)*
M46I	3 (3.33)	*2 (66*.*67)*	1 (1.67)	*1 (100*.*00)*	0 (0)	*0 (0)*
V11I	2 (2.22)	*0 (0)*	1 (1.67)	*0 (0)*	0 (0)	*0 (0)*
V77I	20 (22.22)	*2 (10*.*00)*	13 (21.67)	*1 (7*.*69)*	0 (0)	*0 (0)*
V82I	15 (75.00)	*4 (26*.*67)*	11 (18.33)	*5 (45*.*45)*	1 (2.70)	*0 (0)*
Others: D30N, F53L,G48V,G73C,G73S,I50V,I54V,I64M,K20I,L10F,L76V,M46L,N88S	9 (10.00)	*9 (100*.*00)*	4 (6.67)	*4 (100*.*00)*	0 (0)	*0 (0)*
**RT mutations**^**2**^						
E138A	8 (8.89)	*0 (0)*	4 (6.67)	*0 (0)*	0 (0)	*0 (0)*
V90I	3 (3.33)	*1 (3*.*33)*	3 (5.00)	*2 (6*.*67)*	0 (0)	*0 (0)*
V179D	1 (1.11)	*0 (0)*	3 (5.00)	*2 (6*.*67)*	0 (0)	*0 (0)*
V106M	1 (1.11)	*1 (100*.*00)*	2 (3.33)	*1 (50*.*00)*	1 (2.70)	*1 (2*.*70)*
M41L	1 (1.11)	*1 (100*.*00)*	1 (1.67)	*1 (100*.*00)*	0 (0)	*0 (0)*
M184V	3 (3.33)	*0 (0)*	3 (5.00)	*0 (0)*	0 (0)	*0 (0)*
M184I	1 (1.11)	*1 (100*.*00)*	1 (1.67)	*1 (100*.*00)*	0 (0)	*0 (0)*
K65R	4 (4.44)	*4 (100*.*00)*	2 (3.33)	*2 (100*.*00)*	1 (2.70)	*1 (2*.*70)*
K103S	0 (0.00)	*0 (0)*	2 (3.33)	*2 (100*.*00)*	1 (2.70)	*1 (2*.*70)*
K103N	4 (4.44)	*1 (25*.*00)*	2 (3.33)	*1 (50*.*00)*	0 (0)	*0 (0)*
K101E	3 (3.33)	*0 (0)*	2 (3.33)	*0 (0)*	0 (0)	*0 (0)*
H221Y	0 (0.00)	*0 (0)*	2 (3.33)	*1 (50*.*00)*	0 (0)	*0(0)*
G190A	3 (3.33)	*0 (0)*	1 (1.67)	*0 (0)*	0 (0)	*0 (0)*
E138G	4 (3.33)	*2 (66*.*67)*	1 (1.67)	*0 (0)*	0 (0)	*0 (0)*
Others: A62V, A98G,D67N,E138K,F77L,K101P,K219Q,K70R,L74V,M184I,M230I,P225H,V106A,V108I,V75I,Y181C,Y188H,Y188L	4 (4.44)	*2 (50*.*00)*	15 (25.00)	*10 (6*.*67)*	0 (0)	*0 (0)*

Proportion of minority mutations were calculated as the number of mutations with a frequency under 20% among all existing mutations.

^**1**^ Protease

^**2**^ Reverse transcriptase

### HIV drug resistance mutations at delivery

There were 60 women included in the analysis at the time of delivery. The median viral load was of 34943 copies/ml (IQR: 12891–78116), and it was significantly higher in the women who presented HIVDRM (p = 0.024) (Table 2). Twenty five women (42%) had CD4+ T cell counts < 350 cells/mm^3^. Forty six (82%) women had received ARV drugs for pMTCT and 8 (12%) were on ART for their own health during this pregnancy. Four women (7%) had received neither pMTCT nor ART during this pregnancy. None of the women presenting HIVDRM at delivery transmitted HIV to their infants at first month of age (Table 2). Eight women (13%) presented at least one HIVDRM, 5 (8%) to NNRTIs and 6 (10%) to NRTIs. None of the women had mutations conferring resistance to PIs (Table 3). Among the women presenting HIVDRM to NNRTI, 5 (8%) women had resistant mutations to NVP, 4 (7%) to EFV, 2 (3%) to RPV and 2 (3%) to ETR. Of the women with HIVDRM to NRTI, 3 (5%) had resistant mutations to ddI, 3 (5%) to FTC, 3 (5%) to 3TC, 2 (3%) to TDF, 1 (2%) to ABC and 1 (2%) to d4T (Table 4). The most frequent HIVDR mutations are shown in Table 5.

### HIV drug resistance mutations at enrolment and at delivery

Thirty seven women had plasma samples from both enrolment and delivery available for this analysis. Of these, 5 (14%) developed new drug resistances during this pregnancy, though only one of them (3%) developed HIVDRM to drugs included in the first-line regimen (Table 3). Among those who developed HIVDRM, 4 (80%) had received pMTCT during pregnancy. In addition, among women who developed HIVDRM, none transmitted HIV to their infant at first month of age, while among those women who did not developed HIVDRM, 4 (8%) transmitted HIV to their infants (p = 0.344) (S1 Table).

## Discussion

This is the first study undertaken in Mozambique, -one of the countries with the highest prevalence and incidence of HIV infection in the world-, addressing HIV drug resistance in pregnant women and using NGS. A previous study reported NVP resistance in puerperal women who had received sd-NVP during labor, and all other reports on HIV drug resistance were conducted in either pediatric or adult (non-pregnant) populations [11, 21–23]. This study showed that about 14% of women with detectable viral load at delivery had at least one HIVDRM at enrolment, 10% even before initiating ARV drugs. On the other hand, 3% of the women without HIVDRM at enrolment developed at least one HIVDRM to first line ARV drugs while on pMTCT or ART during pregnancy. Thus, virological failure may occur despite a low proportion of women with pre-existance or acquired HIVDRM, indicating that other factors,—lack of adherence to treatment or prophylaxis, or interruptions of the ANC ‘cascade’, may play a role in explaining the lack of viral suppression at the time of delivery in women receiving ARV drugs during pregnancy.

The prevalence of pre-treatment HIVDRM in this study was 10%, which is higher than that reported (5%) in a previous study carried out in adults in the capital, Maputo, and higher than the 7% found in HIV-infected pregnant women in the south-Africa neighboring region of Kwazulu-Natal [24]. Reports from other countries in the region show prevalences ranging from 5% in Malawi to 12% in Tanzania [24–26]. The prevalence found here exceeds the WHO recommended threshold of 5% transmitted HIV drug resistances at a population-level [9, 27]. Though prior to 2007, most African countries reported transmitted resistance prevalence estimates of <5%, attributed mainly to low ARV drugs coverage, a trend towards increasing prevalence has been observed correlating with time since the initiation of ARV drugs rollout [28, 29]. However, in this study the prevalence of pre-treatment HIVDRM at enrolment may have been over-estimated since only women with detectable viral load at delivery were selected. It is important, to highlight that the use of an ultrasensitive genotyping in this study might have increased the detection of HIVDRM compared to other studies using less sensitive techniques. It is also possible that exposure to ARV drugs for pMTCT in previous pregnancies in study participants could have favored a higher prevalence of resistance compared to the general population. Though differences were not significant, the proportion of multigravidae was higher among women who did not present HIVDRM compared to those who did at enrolment, suggesting that exposure to ARVs in previous pregnancies might not have been frequent among this group. Up to the time of this study, pMTCT in low-income countries consisted of simple regimens, which, combined with lack of virological monitoring or resistance testing, could have fostered the emergence of HIVDRM in pregnancy [30]. Despite the long duration of administration of sdNVP as part of pMTCT in Mozambique, HIV susceptibility to NVP was preserved both at enrolment and at delivery in almost all study women. Although still recommended in the current WHO guidelines, NVP is being phased out from first-line recommended regimens. Similarly, older NRTIs such as the thymidine analogue drugs (AZT and D4T) are being replaced by TDF as part of the NRTI backbone in first-line regimens in resource-limited settings. Our data shows that HIV resistance to regimens used to prevent MTCT is still very limited in the Manhiça district of Mozambique. Moreover, we did not observe a major degree of selection of HIV resistance during pMTCT. Thereby, our findings support current WHO recommendations for the region.

At enrolment, no significant differences were found between women presenting HIVDRM and those who did not. However, among women who had HIVDRM at enrolment there was an increased proportion of adolescents and primigravidae, whom are less likely to have been exposed previously to ARVs, than in those with no HIVDRM. This is in line with our hypothesis that previous exposure to ARV drugs among study participants was low. At the time of delivery, among women that presented HIVDRM, CD4 cell counts were lower and mean viral load was higher compared to women with no HIVDRM. More women transmitted HIV to their infants among those without HIVDRM despite having lower viral loads at the time of delivery. This difference was not statistically significant and may be just due to chance given the small numbers. Other factors favoring MTCT, such as type of delivery, breastfeeding options or uptake of infant prophylaxis for pMTCT were not evaluated. In addition, among the women with HIVDRM at delivery, a higher proportion had been on ART during pregnancy compared to those with no HIVDRM. Poor adherence to ART in those that were already on treatment before pregnancy could explain the presence of drug resistance in this group. In women who received pMTCT, duration of exposure to ARV drugs might have been insufficient to promote the emergence of resistances within the current pregnancy, despite a poor adherence.

In this study, all HIVDRM that emerged during pregnancy were minority mutants. The clinical implications of these minority mutations on developing virological failure in these participants cannot be identified, as all participants had virological failure at delivery as per study inclusion criteria. However, there is evidence suggesting an association between the minority mutations and the risk of virological failure to first-line NNRTI therapy. Similarly, in women in low and middle-income countries (LMIC) previously exposed to NNRTI as regimens to prevent mother-to-child transmission (pMTCT), detection of NNRTI minority mutations also proved to be clinically relevant, also leading to increased risk of virological failure [31–33]. Contrarily, no clinical implications have been reported in the presence of minority resistances when PI-based regimens are used [34]. Further studies should be performed for assessing the impact of current recommended drug-regimens under the Option B+ approach.

The Mozambican national ethics review committee and the Data and Safe Monitoring Board (DSMB) of the trial requested the investigation of the increased viral load and MTCT of HIV in the women who received MQ compared to those who received placebo. This explains the low number of samples in which viral RNA could be amplified, since samples were not stored in the conditions required for this purpose. On the other hand, the limited sample size did not allow making inferences as to whether a possible association between the administration of MQ and the development of HIVDRM during pregnancy, could have explained the observed increased risk of MTCT in the women who received MQ. There is still insufficient information on the potential interactions between antimalarial and ARV drugs [35, 36]. Drug interactions could favor the selection of HIV drug resistance by reducing the plasma concentration of ARV drugs to suboptimal levels. Artemether/lumefantrine for example, has been shown to reduce significantly plasma levels of NVP [37]. In addition, the existing information is mainly limited to artemisinin derivatives [37, 38]. A recent study reported potential pharmacokinetic interactions between MQ-Artesunate (MQ-AS) and lopinavir (LPV/r) in adults, with a reduction in systemic exposure of both drug combinations, suggesting a higher risk of treatment failure for both malaria and HIV infection when the two drugs are co-administered [39]. Another limitation of our study was that data on adherence and on average duration of ARV drugs administration before delivery, which are known to determine viral suppression, was not available. Finally, as the scope of this study was focused on addressing HIVDRM in pregnant women, NGS was not performed in HIV-infected infants. This limits the ability of this study to inform on potential vertical transmission of HIVDRM and should be considered when performing future studies aiming to guide maternal and pediatric care and HIV/AIDS control strategies.

## Conclusions

The latest WHO recommendation of providing all pregnant women with lifelong ART regardless of CD4 count/disease stage (option B+), seems to offer important programmatic and operational advantages and thus, could accelerate progress towards eliminating new pediatric infections. Now that global elimination of HIV pediatric infection is within our reach making NGS accessible for HIV drug resistance surveillance also in LMICs should be a priority considering the increasing HIV-1 resistance and the limited availability of treatment options in these settings. HIV drug resistance surveillance to evaluate the risks for selection of drug resistance under the B+ approach should be performed alongside implementation at the country level.

## Supporting information

S1 Table (DOCX)

S2 Table (DOCX)

S1 Annexe (PDF)
