# Supplementary material for: HIV drug resistance patterns in pregnant women using next generation sequence in Mozambique
Source: PLoS One. 2018 May 9;13(5):e0196451. doi: 10.1371/journal.pone.0196451 (PMC5942837; doi:10.1371/journal.pone.0196451)
Supplement: S1 Table — (DOCX) [file pone.0196451.s001.docx]

**Supplementary table 1. Characteristics at enrolment and at delivery of women who developed HIVDRM during pregnancy**

| **Characteristics** | **All (N=37)** | | **At least one HIVDRM^1^ (N=5)** | | **No HIVDRM^1^** | | **P-value** |
| --- | --- | --- | --- | --- | --- | --- | --- |
|  |  |  |  |  | **(N=32)** | |  |
|  | **n** | **%** | **n** | **%** | **n** | **%** |  |
| **Characteristics at enrolment** | | | | | | | |
| Age^2^ | 25 [23-30] | | 26 [25-29] | | 25 [21-31) | | 0.640 |
| Adolescents^3^ | 6 | 16.22 | 0 | 0.00 | 6 | 18.75 | 0.567 |
| Gestational age, median (IQR)^2,4^ | 21 [18-24] | | 20 [18-26] | | 22 [17-24] | | 0.672 |
| 3^rd^ trimester | 5 | 13.51 | 0 | 0.00 | 5 | 15.63 | 1.000 |
| Primigravidae | 10 | 27.03 | 2 | 40.00 | 8 | 25.00 | 0.597 |
| Number of previous pregnancies^2^ | 2 [0-3] | | 1 [0-3] | | 2 [0-4] | | 0.768 |
| Illiteracy^5^ | 13 | 35.14 | 3 | 60.00 | 11 | 34.38 | 1.000 |
| RPR +^6^ | 5 | 13.51 | 0 | 0.00 | 5 | 15.63 | 1.000 |
| Anemia (Hb<11g/dl) | 28 | 75.68 | 4 | 80.00 | 24 | 75.00 | 1.000 |
| Malnutrition (MUAC^7^<23) | 1 | 2.70 | 0 | 0.00 | 1 | 3.13 | 1.000 |
| HIV-1 RNA (copies/mL)^2^ | 37410 [9081-140129] | | 423000 [107693-427789] | | 34310 [8569-88250] | | 0.050 |
| Detectable HIV-1 RNA ^8^ | 37 | 100.00 | 5 | 100.00 | 32 | 100.00 | 1.000 |
| CD4+ T-cell counts (cell/mm3)^2^ | 01 [251-590] | | 161 [38-593] | | 414 [271-590] | | 0.225 |
| CD4+ T-cell counts <350 cell/mm3 | 14 | 37.84 | 4 | 80.00 | 10 | 31.25 | 0.057 |
| Previous antiretroviral therapy | 9 | 24.32 | 1 | 20.00 | 10 | 31.25 | 0.232 |
| **Characteristics at delivery** | | | | | | | |
| IPTp treatment with MQ^9^ | 16 | 43.24 | 3 | 60.00 | 13 | 40.63 | 0.634 |
| HIV-1 RNA (copies/mL)^2^ | 37925 [16236-88998] | | 166741 [144722-249909] | | 35389 [15561-72760] | | 0.068 |
| CD4+ T-cell counts (cell/mm3)^2^ | 401 [251-590] | | 161 [38-593] | | 414 [271-590] | | 0.225 |
| CD4+ T-cell counts <350 cell/mm3 | 14 | 37.84 | 3 | 60.00 | 11 | 34.38 | 0.263 |
| Antiretroviral drugs during pregnancy | | | | | | | |
| -             PMTCT^10^ | 33 | 89.19 | 4 | 80.00 | 1 | 3.13 | 0.456 |
| -             ART^11^ | 2 | 5.41 | 1 | 20.00 | 2 | 6.25 |  |
| -        None | 2 | 5.41 | 0 | 0.00 | 29 | 87.88 |  |
| MTCT ^12,13^ | 4 | 14.29 | 0 | 0.00 | 4 | 15.38 | 0.344 |

^1^ HIV drug resistance mutation ^2^ Median [IQR] ^3^ Age<19 years ^4^ Gestational age calculated from fundal height ^5^ Not being able to read and/or write ^6^ Rapid plasma regain test ^7^ Middle upper arm circumference ^8^ HIV-1 RNA>400 copies/ml ^9^ Intermittent treatment of malaria in pregnancy ^10^ Prevention of mother to child transmission: AZT monotherapy during pregnancy + sigle dose NVP intrapartum + AZT and 3TC one week post-partum ^11^ Antiretroviral therapy ^12^ Mother to child transmission of HIV ^13^ Data available only from 28 infants
