# Supplementary material for: HIV drug resistance patterns in pregnant women using next generation sequence in Mozambique
Source: PLoS One. 2018 May 9;13(5):e0196451. doi: 10.1371/journal.pone.0196451 (PMC5942837; doi:10.1371/journal.pone.0196451)
Supplement: S2 Table — (DOCX) [file pone.0196451.s002.docx]

**Table S2 MTCT in women with samples at delivery, by maternal HIVDRM, PMTCT intervention and sex of infants**

| **Variables** | | **MTCT** | |
| --- | --- | --- | --- |
|  |  | **Positive  *(N=4)*** | **Negative  *(N=43)*** |
| HIVDRM | | 0 (0%) | 2 (4.7%) |
| Maternal PMTCT option | PMTCT | 2 (50.00%) | 37 (86.05%) |
|  | HAART | 1 (25.00%) | 4 (9.30%) |
|  | NONE | 2 (25.00%) | 2 (4.7%) |
| Sex (Male) | | 1 (25.00%) | 22 (51.16%) |
