# Supplementary material for: HIV drug resistance patterns in pregnant women using next generation sequence in Mozambique
Source: PLoS One. 2018 May 9;13(5):e0196451. doi: 10.1371/journal.pone.0196451 (PMC5942837; doi:10.1371/journal.pone.0196451)
Supplement: S1 Annexe — (PDF) [file pone.0196451.s003.pdf]

15/07/2014
Tomei conhecimento e por favor fazer copia urgente para Dra Clara, Maria e Dr. Inacio
[illegible]

REPÚBLICA DE MOÇAMBIQUE

MINISTÉRIO DA SAÚDE

COMITÉ NACIONAL DE BIOÉTICA PARA A SAÚDE
IRB00002657

Exmo Senhor
Dr. Eusébio Macete
CISM

Ref: 209/CNBS/2014

Data 03 de Julho de 2014

Assunto: Parecer do Comité Nacional de Bioética para saúde (CNBS) sobre: *"Análise de resistência aos anti-retrovirais nas participantes no estudo MiPPAD"*

No dia 23 de Junho de 2014, o Comité Nacional de Bioética para Saúde (CNBS) avaliou o pedido de autorização para a realização da ***"Análise de resistência aos anti-retrovirais nas participantes no estudo MiPPAD"***; sobre o mesmo o CNBS chegou a seguinte conclusão:

Não havendo nenhum inconveniente de ordem ética que impeça a continuação do estudo, o CNBS dá a sua devida autorização.

Todavia, o CNBS recomenda aos investigadores que o mantenham informado do decurso do estudo.

Com as nossas mais cordiais saudações.

O Presidente

João Schwalbach

Dr. João Fernando Lima Schwalbach

ENDEREÇO:
MINISTÉRIO DA SAÚDE
C. POSTAL 264
Av. Eduardo Mondlane/Salvador Allende
MAPUTO – MOÇAMBIQUE

Telefones: 430814/427131(4)
Telex: 6-239 MISAU MO
FAX: 258 (1) 426547
258 (1) 33320

Dña. Begoña Gómez Pérez, Secretaria del Comité Ético de Investigación Clínica del Hospital Clínic i Provincial de Barcelona,

CERTIFICA:

Que este Comité, con fecha 09/10/08, ha evaluado la propuesta del promotor para que se realice el ensayo clínico código de protocolo IP.07.31080.002 **titulado "Evaluation of the safety and efficacy of mefloquine as intermittent preventive treatment in pregnacy". Versión 01 de 25/09/08; CI: versión de 25/09/08**, y considera que:

. Se cumplen los requisitos necesarios de idoneidad del protocolo en relación con los objetivos del estudio y están justificados los riesgos y molestias previsibles para el sujeto.

. La capacidad del investigador y los medios disponibles son apropiados para llevar a cabo el estudio.

. Son adecuados tanto el procedimiento para obtener el consentimiento informado como la compensación prevista para los sujetos por daños que pudieran derivarse de su participación en el ensayo.

. El alcance de las compensaciones económicas previstas no interfiere con el respeto a los postulados éticos.

Que este Comité acepta que dicho ensayo clínico sea realizado en el Centro de Investigaçao em Saúde de Manhiça (CISM), Mozambique por la **Dra. Menéndez Santos, Clara** como investigador principal, debiendo ser comunicado a dicho Comité Ético todo cambio en el protocolo o acontecimiento adverso grave.

Lo que firmo en Barcelona, a 17 de octubre de 2008

CLÍNIC
BARCELONA
Hospital Universitari
COMITÈ ÈTIC
INVESTIGACIÓ CLÍNICA

CIF - G-08431173

HOSPITAL CLÍNIC I PROVINCIAL DE BARCELONA
Villarroel, 170 - 08036 Barcelona (España)
Tel. 93 227 54 00 Fax 93 227 54 54
www.hospitalclinic.org

852
